# Anatomical and Embryological Development of the Chick Cerebrum in Different Embryonic Periods

**DOI:** 10.1002/vms3.70124

**Published:** 2025-01-10

**Authors:** Muhammet Lutfi Selcuk, Fatma Kayikci

**Affiliations:** ^1^ Faculty of Health Sciences, Department of Physiotherapy and Rehabilitation Karamanaoglu Mehmetbey University Karaman Turkey; ^2^ Faculty of Health Sciences, Department of Nutrition and Dietetics Karamanoglu Mehmetbey University Karaman Turkey

**Keywords:** cerebrum, chick embryo, developmental embryology, stereology

## Abstract

The objective of this study is to assess the embryological and morphometric development of the chick cerebrum during specific incubation periods. The cerebrums of 24 Babcock White Leghorn chicks, six each from the 10th, 13th, 16th and 21st days of the incubation period, were used in the study. After removing the heads of fixed embryos from the upper edge of the atlas, the brains were taken out of the cranial cavity. Morphometric measurements were performed on the removed brains, and paraffin blocks were prepared following the routine histological procedure. Sections 5 µm thick were taken from the blocks, with an additional 10 µm thick section taken every 50th section. The slides were then stained using Crossmon's triple stain and Klüver–Barrera staining methods and photographed. The sectional images were transferred to the ImageJ programme, brain volume was calculated using stereological methods, and histological measurements were performed. The development of brain parts in selected embryonic periods was examined in detail, focusing on anatomical and histological aspects. According to the results, it was determined that all measured parameters, except the third ventricle width, increased and were statistically significant (*p* < 0.05). It is believed that the findings of this study will enhance the understanding of the region's anatomy. The new morphometric data can serve as reference data in neurotoxicity and embryotoxicity studies.

## Graphical abstract

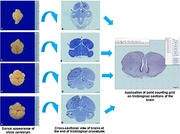

## 1 Introduction

The tubular embryonic chick brain is the earliest organ to form during the embryonic period (Chen et al. 2016). The egg expelled by ovulation has an embryonic formation consisting of epiblast and hypoblast layers. The cells that are guided by the hypoblast and will form the forebrain area move towards the anterior end of the primitive line (Wittler and Kessel 2004). As incubation progresses, sulcus neuralis is formed as a second groove in the anterior part of the nodus primitivus, which is formed as a result of the thickening of the tip of the primitive line. This structure closes and forms the neural canal, which constitutes the origin of the central nervous system (Hassa 1985; Lawson, Anderson, and Schoenwolf 2001).

In the early embryonic period, the constrictions formed along the rostrocaudal axis form the prosencephalon (forebrain), mesencephalon (midbrain) and rhombencephalon (hindbrain) (Garcia et al. 2017). As incubation progresses, the prosencephalon develops as a protrusion from the anterior end of the neural tube; it is divided into two parts: telencephalon and diencephalon (Puelles et al. 2012; Sadler 2006). Then, the telencephalon develops into the right and left cerebral hemispheres through extensive growth and divisions (Garcia et al. 2019). When the neural tube closes, embryonic cerebrospinal fluid is secreted into all brain cavities (Garcia et al. 2017).

It is stated that the brain of birds is larger compared to other vertebrates (Abid and Al‐Bakri 2017). Some researchers state that chicken embryo development between the incubation 14th and 21st days is similar to the third trimester of human embryonic development, and the chicks have a well‐developed brain at hatching (Nishigori et al. 2013; Zhou et al. 2015). Abid and Al‐Bakri (2017) report that the quail brain is a large‐sized, triangular‐shaped organ consisting of prosencephalon, mesencephalon and rhombencephalon. It has folds formed by superficial shallow groove.

The dorsal part of the prosencephalon, which is covered with meninges, appearing as sacs and larger in birds, is called the telencephalon (cerebral hemispheres), and its ventral part is called the diencephalon. Ninety percent of the neurones in the central nervous system are located in the telencephalon (Aspinall and Cappello 2015; Bölükbaş and Öznurlu 2023; Gautam et al. 2020). In birds, the telencephalon consists of two different regions: pallium and subpallium. The pallium includes the dorsolateral corticoid area and dorsal ventricular ridge areas (Abid and Al‐Bakri 2017; Gautam et al. 2020; Jarvis 2009; Kuenzel 2018). The pallium covers approximately 75% of the telencephalon in adult birds (Iwaniuk and Hurd, 2005; Shepherd 1994). The dorsal ventricular ridge areas that control wing movement and body position are underdeveloped in pigeons, quails and chickens (Jarvis et al. 2005). The subpallium region includes the striatum that contains neuronal fibres and the pallidum, which is the deepest part of the cerebrum (Abid and Al‐Bakri 2017; Gautam et al. 2020; Jarvis 2009). The subpallium, which is the main centre of the extrapyramidal motor system, is called the basal ganglia. In addition, it has been reported that the striatum, which resembles the neocortex region of mammals, contains basal ganglia and nerve fibre bundles (Puelles et al. 2000; Skimizu 2009; Weithers 1992). The mesencephalon is the part of the brain where the large optic lobes are located. The dorsal part of these lobes is called the optic tectum, and the ventral part is called the tegmentum (Abid and Al‐Bakri 2017). Well‐developed optic lobes are an indicator of good vision in birds (Batah, Ghaje, and Aziz 2012; Gautam et al. 2020). Rhombencephalon is the rhomboid‐shaped structure in the back part of the brain (Abid and Al‐Bakri 2017).

Coelho et al. (2018) state that brain diseases are characterized by synaptic loss or neuronal degenerations. Regional brain volume changes or atrophy rates are considered key markers of disease progression in the clinic (Selcuk and Bahar 2014; Heggland et al. 2015). In addition, histological and micro‐anatomical methods are frequently used to distinguish substantia grisea and substantia alba in the mammalian brain, calculate volume and volume ratios, and distinguish and determine cell types in the brain (Bahar, Bolat, and Selcuk 2013; Bolat 2018).

In developmental embryonal studies, the fact that chicken embryos can be easily manipulated and do not have a placental barrier is very important for better understanding organ morphogenesis. Although the mechanisms in the embryonic development of the brain have been examined to date, it is known that there are a number of processes that are still not fully clarified. The aim of this study is to determine the embryological and morphometric development of the embryonic chick cerebrum in certain incubation periods. It is planned to obtain new information about the avian cerebrum by following the embryological development in cellular and anatomical aspects. It is envisaged that the data obtained will be used as a reference source for future neurotoxicity and embryotoxicity studies.

## 2 Materials and Methods

### 2.1 Materials

In the study, the cerebrum of 24 Babcock White Leghorn chicks, 6 from each of the 10th, 13th, 16th and 21st days of incubation according to the Hamburger and Hamilton Scale (HH scale), was used (Hamburger and Hamilton 1951). On the determined embryonic days, the eggs were randomly opened and evaluated in terms of brain development until six live embryos were obtained from each group, and the embryos were fixed in a 10% buffered formol saline (pH 7.4) solution for 15 days at room temperature.

### 2.2 Morphometric Measurements

After fixation, the embryo heads were separated from the upper edge of the atlas, and the brains were removed from the cranial cavity. The cerebrum's weight was measured with a precision scale. The length of the cerebrum, width of the cerebrum, length of the optic tectum and width of the optic tectum were measured with the digital caliper. The relative weight of the cerebrum (RCW) was calculated by dividing the weight of the cerebrum by the weight of the embryo. To obtain the density of chick cerebrum (g/mL), its weight was divided by its volume. In examining cerebrum development on determined embryonic days, the thickness of optic tectum wall, width of the optic tectum ventricle, thicknesses of the optic tectum layers and width of the third ventricle of each embryo were measured from six different regions, and average values were taken.

### 2.3 Histological Processing of Cerebrum

The cerebrum, fixed in a 10% neutral buffered formalin solution, was removed from its peripheral tissues and placed in tracking cassettes. Then, these cerebrums were washed in running water overnight. Paraffin blocks of the samples for routine histological follow‐up were prepared. Three sagittal serial sections (5 µm thickness) were taken from each block at regular intervals for histological examination using a rotary microtome. For cerebrum volume calculation, 10 µm thick sections were taken every 50 sections to a slide coated with poly‐l‐lysine. Crossmon's trichrome staining (Crossmon 1937; Selçuk and Tıpırdamaz 2020) and Klüver–Barrera staining (Klüver and Barrera 1953; Selçuk and Çolakoğlu 2020) were applied to these sections. Then, these preparations were covered with entellan. Digital images of the necessary areas of the preparations, which were evaluated under a camera‐attached microscope (Leica DM‐2500 attached to a DFC‐320 digital camera), were recorded. The ImageJ analysis programme was used for cerebrum measurements.

### 2.4 Volume Calculations in Cerebrum

**FIGURE 1:**
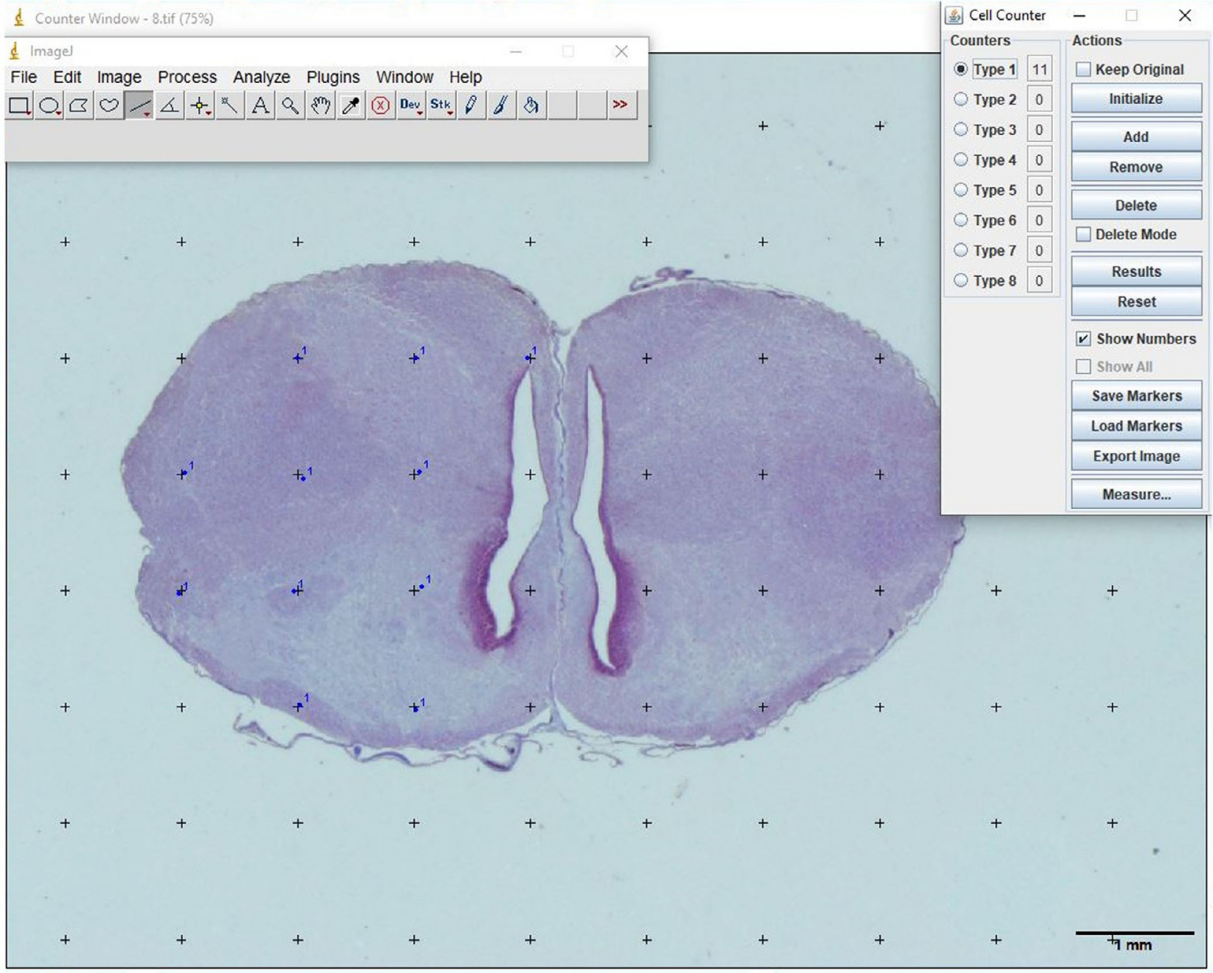
Application of point counting grid on histological sections of the brain.

The grid function of the ImageJ programme was used in volume calculations. First, ImageJ was calibrated, and a point‐counting grid (*d* = 1 mm) was applied to the cross‐sectional images (Figure 1). The counting process was carried out by counting the points falling on the cerebrum.

The formula used to estimate the volumes was *V* = *a*(*p*) × Σ*p* × *t* (Mayhew and Gundersen 1996). In this formula, *V* represents the volume of the cerebrum, *a*(*p*) was the area of one point on the grid (1 mm^2^ in this study), Σ*p* was the sum of the points on the structure of interest and *t* was the section thickness (Selcuk and Bahar 2014). The coefficient of error (CE) was calculated according to the study of Gundersen et al. (1999).

### 2.5 Statistical Analysis

In the study, the analysis of morphometric data obtained from cerebrum was performed using the SPSS 21.0 statistical package programme. The normal distribution of the variables was confirmed through analytical methods such as the Kolmogorov–Smirnov/Shapiro–Wilk tests, as well as through a histogram and probability graphs. One‐way ANOVA test was used for statistical comparisons of data obtained from the groups, where the *p* value was significant, and pairwise post‐hoc comparisons between statistically significant results were made using the Tukey test. Data are expressed as means ± standard deviation (SD). *p* < 0.05 was accepted as statistically significant.

## 3 Results

### 3.1 Macroscopical and Anatomical Evaluations

In the macroscopic examination of embryonic chick brains, it was noted that the chick brain gradually grew larger as following incubation days, changed from an oval‐triangle‐shaped appearance to oval‐like, and the folds formed from the shallow grooves on its surface increased considerably.

**FIGURE 2:**
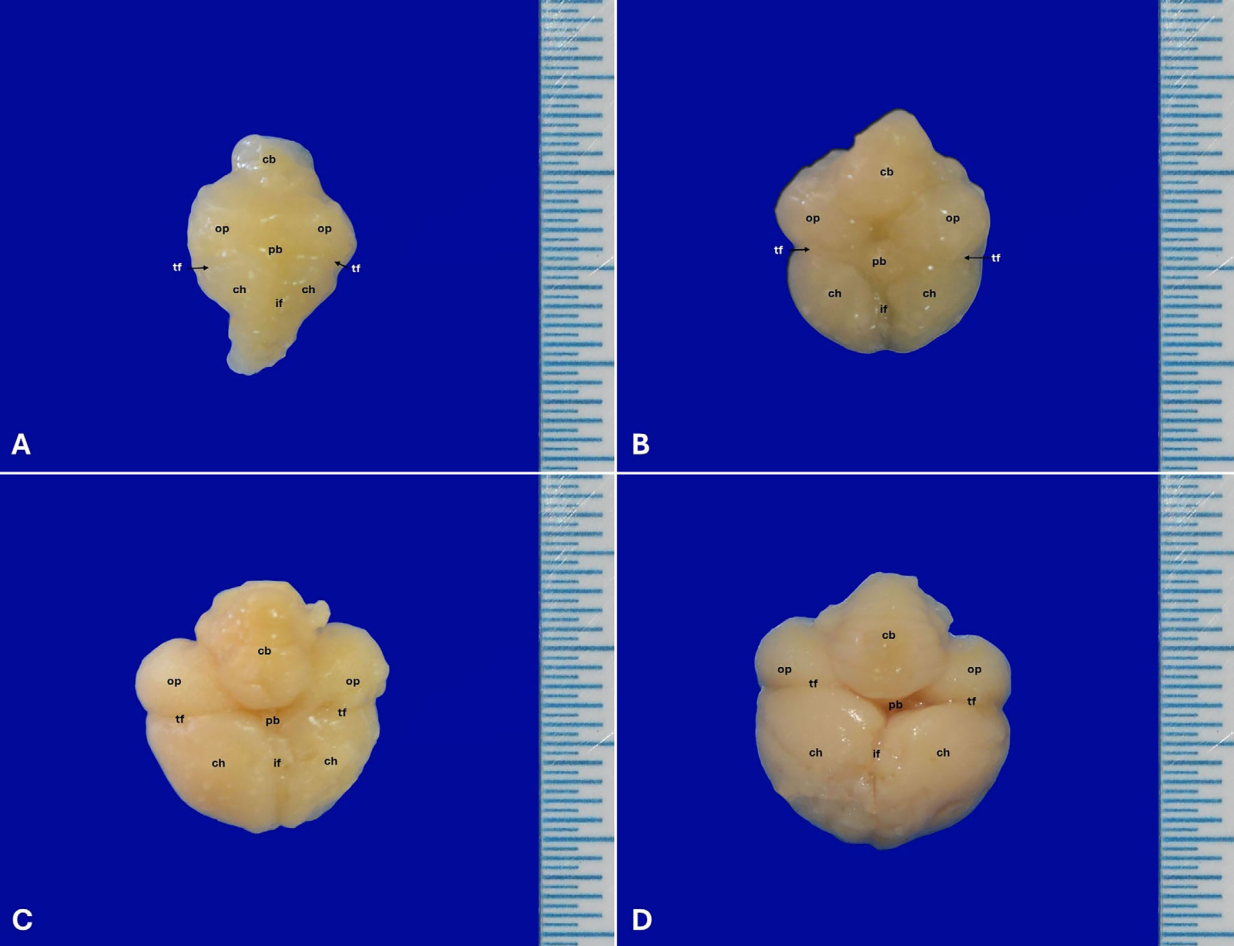
Dorsal appearance of chick cerebrum: (A) 10th day, (B) 13th day, (C) 16th day and (D) 21st day. cb: cerebellum, ch: cerebral hemisphere, if: interhemispheric fissure, op: optic lobe, pb: pineal body, tf: transverse fissure.

The distinction among prosencephalon, mesencephalon and rhombencephalon, which constitute the cerebrum, could be easily made in the chicken embryo on the 10th day of incubation. The cerebral hemispheres, which were covered externally by the pia mater and formed the telencephalon, were separated from each other by the cerebral fissure. The transverse cerebral fissure separating the cerebrum and cerebellum was also seen in the embryos. Cerebral hemispheres on Day 10 were quite small compared to other cerebrum parts. On other determined incubation days, developmental changes in the embryonic chick cerebrums were easily observed as the incubation period progressed. The cerebral hemispheres at hatching were significantly larger than those on the 10th day. On the 21st day, it was determined that chick cerebrums had a normal cerebrum appearance in terms of both dimensional growth and oval shape (Figure 2). The optic tectums were shaped as symmetrically oval‐like protrusions on the ventrolateral side of the mesencephalon. It was observed that these structures develop in the following incubation days. Some morphometric data of the chick cerebrum are given in Table 1. Statistically significant differences were observed in all measured parameters, except for third ventricle width (*p* < 0.05).

**TABLE 1:** Some morphometric data of the chick cerebrum in different incubation periods.

Parameters	Day of incubation (*n* = 6)			
	10th	13th	16th	21st
Embryo weight (g)	3.04 ± 0.16^a^	8.79 ± 0.25^b^	22.57 ± 2.02^c^	40.83 ± 3.29^d^
Cerebrum weight (g)	0.17 ± 0.02^a^	0.34 ± 0.03^b^	0.37 ± 0.02^c^	0.48 ± 0.01^d^
Cerebrum volume (mL)	0.137 ± 0.007^a^	0.183 ± 0.048^a^	0.426 ± 0.139^b^	0.532 ± 0.072^b^
Coefficient of error (CE)	0.0256	0.0338	0.0302	0.0228
Density of chick cerebrum (g/mL)	1.22 ± 0.18^a^	1.96 ± 0.64^b^	0.94 ± 0.26^a^	0.92 ± 0.11^a^
Relative weight of the cerebrum	0.055^a^	0.038^b^	0.017^c^	0.012^c^
Length of the cerebrum (mm)	6.47 ± 0.44^a^	7.93 ± 0.68^a^	8.71 ± 1.74^b^	9.60 ± 0.40^b^
Width of the cerebrum (mm)	8.29 ± 0.36^a^	9.440 ± 0.80^a^	10.99 ± 0.70^b^	11.92 ± 0.95^b^
Craniocaudal length (mm)	4.50 ± 0.55^a^	8.67 ± 0.75^b^	9.83 ± 0.52^b^	10.67 ± 1.13^c^
Thickness of optic tectum wall (µm)	913.47 ± 158.49^a^	935.26 ± 140.90^a^	1177.73 ± 50.05^b^	1345.15 ± 162.35^b^
Width of the optic tectum ventricle (µm)	588.03 ± 174.65^a^	535.16 ± 215.69^a^	428.35 ± 169.97^a^	754.04 ± 171.50^b^
Width of third ventricle (µm)	1147.90 ± 207.06	903.52 ± 381.88	889.45 ± 281.44	1094.75 ± 56.76
Optic tectum length (mm)	2.89 ± 0.14^a^	3.24 ± 0.32^a^	4.54 ± 0.58^b^	4.04 ± 0.56^b^
Optic tectum width (mm)	2.27 ± 0.17^a^	2.22 ± 0.25^a^	2.94 ± 0.40^b^	3.37 ± 0.27^b^
Width of stratum opticum (µm)	56.25 ± 9.50^a^	67.17 ± 7.06^a^	67.07 ± 18.03^a^	124.31 ± 23.43^b^
Width of stratum griseum superficial (µm)	158.59 ± 26.04^a^	204.95 ± 29.49^a^	243.89 ± 76.37^b^	258.18 ± 41.52^b^
Width of stratum griseum central (µm)	89.05 ± 12.52^a^	110.34 ± 53.81^ab^	120.59 ± 21.98^ab^	162.36 ± 25.31^b^
Width of stratum album centrale (µm)	480.75 ± 34.61^a^	508.17 ± 47.99^a^	636.67 ± 110.88^a^	768.89 ± 242.22^b^

^1^ Different letters in the same row ^(a, b, c, d)^ indicate significant differences (*p* < 0.05).

### 3.2 Embryonic Development of Cerebrum

**FIGURE 3:**
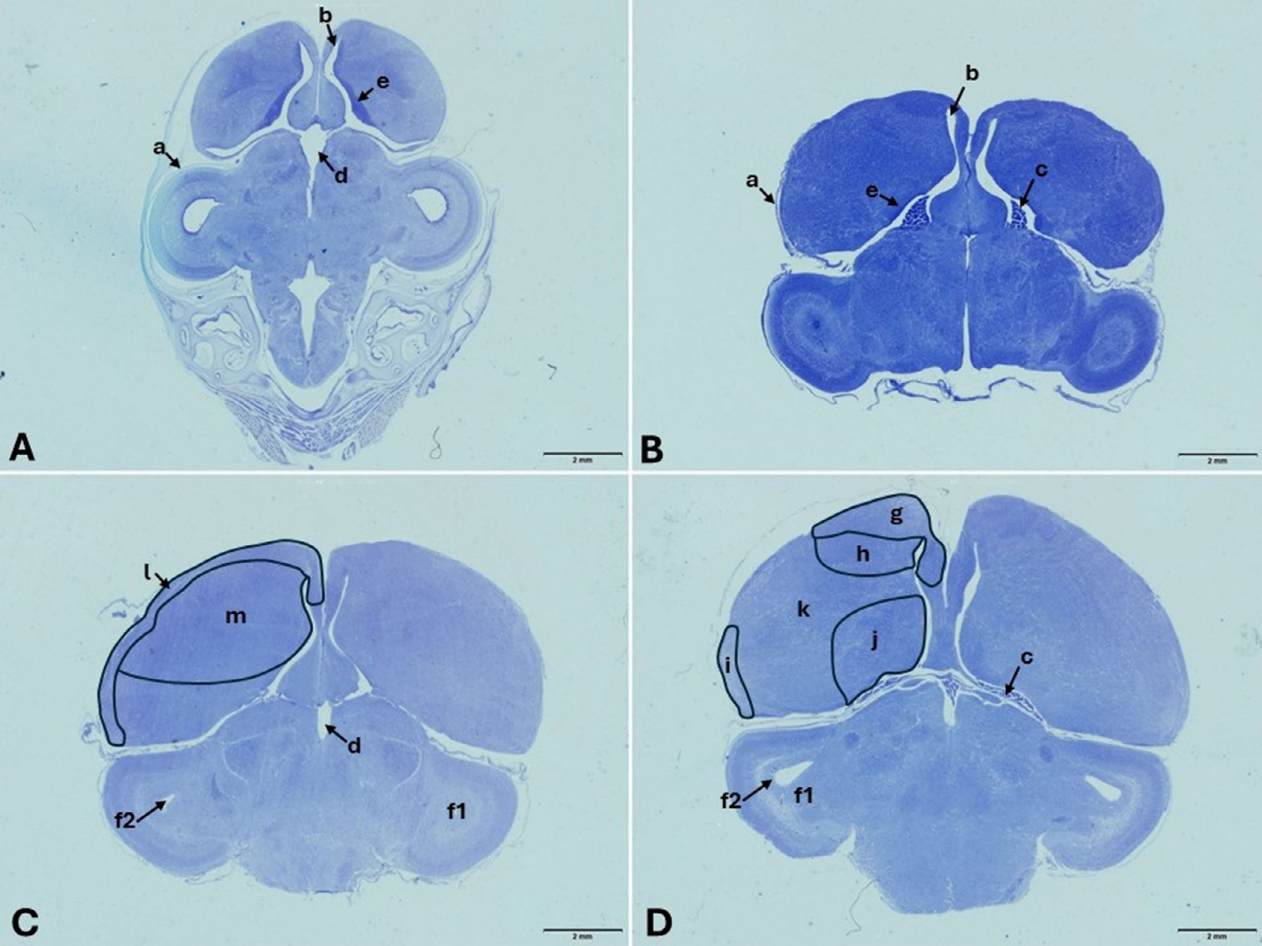
Embryonic development of cerebrum in different embryonic periods: (A) 10th day, (B) 13th day, (C) 16th day and (D) 21st day. a: pia mater, b: lateral ventricle, c: choroid pleksus, d: third ventricule, e: neuroepithelium, f1: optic tectum, f2: optic ventricle, g: hippocampal area, h: mezopallium, I: piriform cortex, j: subpallium, k: nidopallium, l: dorsolateral corticoid area m: dorsal ventricular ridge areas, Crossmon's trichrome staining.

**FIGURE 4:**
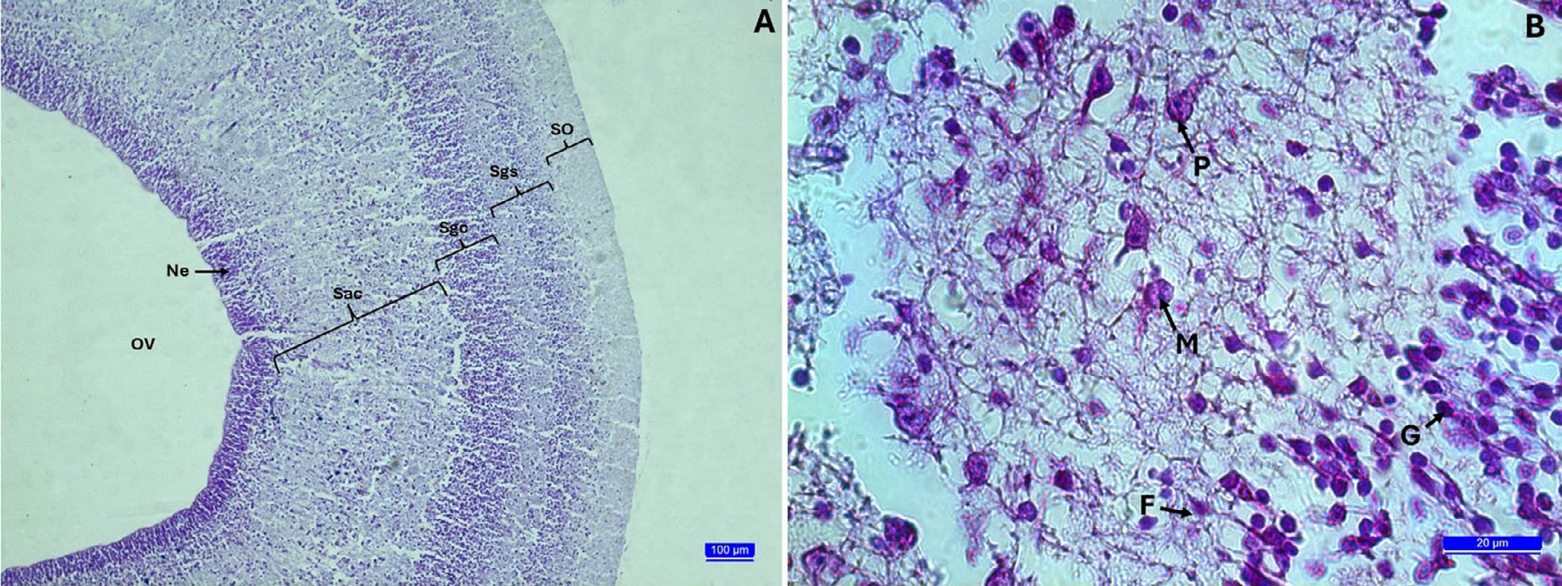
(A) The section from embryonic development of optic tectum on the 10th incubation day. SO: stratum opticum, Sgs: stratum griseum superficial, Sgc: stratum griseum central, Sac: stratum album central, Ne: neuroepithelium, OV: optic ventricle, Crossmon's trichrome staining (10×). (B) A section from the stratum griseum centrale. P: pyramidal cell, M: multipolar cell, F: fusiform cell, G: glial cell, Crossmon's trichrome staining (100×).

In the microscopic examination, it was observed that chick cerebrums were surrounded by pia mater throughout all determined incubation periods. Microscopically, while an oval‐triangle‐shaped cerebrum structure was seen on the 10th day of incubation (HH scale, Stage 36), the cerebrum sections prosencephalon, mesencephalon and rhombencephalon could also be easily differentiated. There were primitive telencephalic vesicles that would shape the telencephalon part of the prosencephalon, containing the cerebral hemisphere outlines and the diencephalon. Large and crescent‐shaped lateral ventricles were located around these vesicles on both sides. In addition, the third ventricle, located between the diencephalon and rhombencephalon, was easily detected. The prosencephalon was smaller compared to the mesencephalon and rhombencephalon. On the 10th day, the presence of primitive choroid plexus in the lateral ventricles was noted. In the parenchymal tissue of the telencephalon, the pallium and subpallium regions are primitive. The dorsolateral corticoid area and dorsal ventricular ridge forming the pallium were shaped. In the cerebrum areas adjacent to the ventricles, the neuroepithelium structure still existed and was quite prominent. It was observed that neuroepithelial cells were quite dense in the parenchymal tissue. There were occasionally large pyramidal cells in the nidopallium region of the dorsal ventricular ridge (Figure 3A). Optic tectums were quite large during this incubation period. In these structures in the mesencephalon, the cellular layers began to take shape from outside to inside as stratum opticum, stratum griseum superficiale and stratum griseum centrale. Stratum griseum periventriculare and stratum fibrosum periventriculare could not be distinguished from stratum album centrale during this incubation period. The optic ventricle wall was lined with neuroepithelium cells (Figure 4A).

The cerebrum structure on the 13th day of incubation (HH scale, Stage 39) was at a more advanced stage than that on the 10th day. It was noted that the primitive cerebral hemispheres were quite well developed and enlarged compared to the previous incubation day. As a result of this situation, the oval‐triangle‐shaped brain appearance began to be replaced by an oval‐shaped brain. The choroid plexus in the lateral ventricles was quite well developed on this embryonic day. The perivascular space formed by the arteriole entering the brain parenchyma and the surrounding pia mater was visible during this period. Compared to the 10th day, the crescent‐shaped lateral ventricles were reduced in size. The cellular layers in the optic tectum continued to develop. The neuroepithelium adjacent to the ventricles still existed in some places (Figure 3B). The innermost two layers of the optic tectum were still not clearly distinguishable from the stratum album centrale. During this period, the optic ventricle wall was still lined with neuroepithelium cells.

When the chick cerebrums on the 16th day of incubation (HH scale, Stage 42) were examined, it was seen that the prosencephalon had grown considerably, and the cerebrum had taken a completely oval shape. The pia mater, which surrounds the cerebrum from the outside, penetrated into the brain parenchyma and greatly developed the choroid plexus network in the cerebrum ventricles. The cerebrum parenchyma was rich in blood vessels, and vascularization was developed. It was determined that the cerebral hemispheres were getting closer to each other as the lateral ventricles became smaller. Cerebrum parts could be easily distinguished from each other. Pallium and subpallium areas could be easily distinguished in the cerebrum. Although pyramidal cells were frequently encountered in the hippocampal area, piriform cortex, mesopallium and nidopallium areas that make up the pallium, these cells were less numerous in the subpallium area (Figure 3C). The development of four cellular layers in the optic tectum was quite evident. The stratum griseum superficiale contained fusiform cells and multipolar cells, as well as abundant glial cells. Although pyramidal cells were the majority in the stratum griseum centrale, there were also multipolar cells, fusiform cells and glial cells this layer. The innermost three layers of the optic tectum were not clearly distinguishable. During this period, the optic ventricle was covered with a layer of cuboidal ependymal cells (Figure 4B).

**FIGURE 5:**
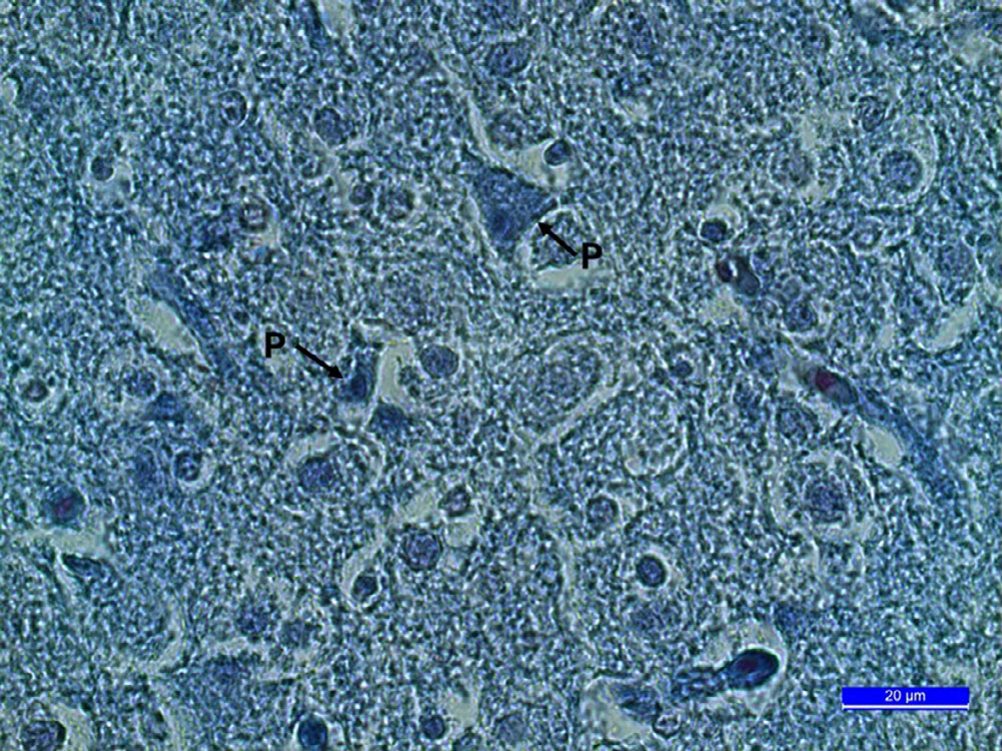
Pyramidal cell structure in the nidopallium region. Klüver–Barrera staining (100×).

At hatching (HH scale, Stage 46), the embryonic chick cerebrum resembled an adult chick cerebrum with its oval shape, well‐developed cerebral hemispheres and choroid plexus. Ventricular spaces were quite narrow. The pallium and subpallium regions were more easily distinguishable than the 10th day (Figure 3D). Large pyramidal cells were quite evident in the nidopallium surrounding the lateral ventricles and were easily seen (Figure 5). Although the development of four cellular layers in the optic tectum became evident, stratum griseum periventriculare and stratum fibrosum periventriculare were not clearly distinguishable from stratum album centrale.

## 4 Discussion

Cerebrum research in poultry, which is widely used for consumption and research purposes, is very important in terms of gaining information on many issues such as animal welfare, anatomical development and neural functions (Gautam et al. 2020). It is frequently preferred in neurotoxicity and embryotoxicity studies because it allows us to see the long‐term effects of chicken embryos, which do not have a placental barrier that may occur because of a single exposure to exogenous and endogenous factors (Yadav et al. 2022). In this study, chick cerebrum development was divided into specific embryonic periods and examined anatomically and histologically with volumetric and diameter measurements.

Triangular/oval‐shaped brains in hatched broiler chicks were found to have an average length and width of 10.09 ± 0.33 mm and 16.84 ± 0.07 mm in 14‐day‐old embryos, and 12.53 ± 0.15 mm and 17.19 ± 0.22 mm in 21‐day‐old embryos, respectively (Gautam et al. 2020). Faisal et al. (2023) found the brain length, brain width and brain density in 14‐day‐old chick embryos as 15.47 ± 0.708 mm, 14.14 ± 1.005 mm and 1.33 ± 0.143 g/mL, respectively. Although the cerebrum length of adult local chickens was 17.45 ± 0.34 mm, the cerebrum width was measured as 20.74 ± 0.78 mm (Batah, Ghaje, and Aziz 2012). In the presented study, brain length was found to be 7.93 ± 0.68 mm, brain width was 9.440 ± 0.80 mm and brain density was 1.96 ± 0.64 g/mL in 13‐day‐old chick embryos. In 21‐day‐old chick embryos, these values were 9.60 ± 0.40 mm, 11.92 ± 0.95 mm and 0.92 ± 0.11 g/mL, respectively. The differences detected between the data obtained from the presented study and the researchers' data were attributed to chick breeds in the studies and incubation days when the embryos were taken.

A study conducted using MRI imaging technique reported that the chick brain grows most rapidly between the 12th and 17th days of incubation. They also state that the chick brain grows non‐linearly during incubation and that flexible points appear on the line on the 6th, 12th, 14th and 17th days of incubation (Zhou et al. 2015). The findings in the present study were similar. Atallah et al. (2021) found the embryonic brain weight to be 0.846 ± 0.002 g on the 20th day of incubation, whereas Faisal et al. (2023) measured the same value as 0.2783 ± 0.010 g on the 14th day of incubation. In the present study, this value was 0.34 ± 0.03 g on the 13th day and 0.48 ± 0.01 g on the 21st day.

Zhou et al. (2015) found that the brain volume was 458.48 ± 17.68 mm^3^, 695.89 ± 14.37 mm^3^, 881.36 ± 11.36 mm^3^ and 1117.96 ± 31.45 mm^3^ on the 12th, 15th, 17th and 20th days of incubation, respectively. They reported that the embryonic chick brain contains a large amount of cerebrospinal fluid in the early developmental stage (5th–12th embryonic days), and cortical thickness increases on 14th–20th days. In 14‐day‐old chick embryos, Faisal et al. (2023) found brain volume, and width of the third ventricle as 0.2243 ± 0.021 mL and 454.77 ± 9.61 µm, respectively. In the presented study, the cerebrum volume was found to be 0.137 ± 0.007 mL on the 10th day, 0.183 ± 0.048 mL on the 13th day, 0.426 ± 0.139 mL on the 16th day and 0.532 ± 0.072 mL on the 21st day of incubation. The width of the third ventricle was also found to be 903.52 ± 381.88 µm on the 13th day. Our study was consistent with the study of Zhou et al. (2015). Our study supports the presence of a large amount of cerebrospinal fluid in the early period, as evidenced by the fact that the third ventricle width did not change. It was thought that the third ventricle width remained constant due to the large amount of cerebrospinal fluid and the development of regional tissues. However, our study was incompatible with Faisal et al. (2023). It was thought that the observed difference was due to breed differences.

Toledo Fonseca et al. (2013) stated that optic vesicles were first observed on the 4th embryonic day. The optic lobes were symmetrically shaped as sphere‐oval projections located in the ventro‐lateral part of the midbrain (Gautam et al. 2024). Faisal et al. (2023) detected the width optic lobe cavity and the thickness of the optic lobe wall as 371.004 ± 12.34 µm and 732.73 ± 20.00, respectively. The optic ventricle epithelium consists of cuboidal ependymal cells. It is reported that the optic tectum consists of six layers with various neurone types (pyramidal, multipolar and fusiform), and the thickness of these layers increases with age. The stratum fibrosum periventriculare was not well separated from the stratum griseum periventriculare, up to the 7th day in post‐hatching broiler chickens (Gautam et al. 2024). Our findings were consistent with those of the researchers.

It has been reported that telencephalic vesicles develop in the early stages of embryonal development and that the telencephalon, cerebellum and brainstem can be distinguished on the 9th day of incubation. The cerebrum is divided by a median fissure into pear‐shaped right and left cerebral hemispheres by a longitudinal slit. The cerebral hemispheres, whose rostral end is narrow and whose caudal end is gradually widening, are separated from the cerebellum by a narrow and transverse fissure, and this slit in the cerebrum is closed by the meninges. The cerebral hemispheres, the largest part of the prosencephalon, contain 90% of the neurones in the central nervous system (Abid and Al‐Bakri 2017; Gautam et al. 2020). In this study, similar findings were obtained.

According to Atay et al. (2020), the various cellular processes in chicks result in the shaping of different brain parts that can be easily identified on the 15th, 18th and 21st days of incubation. Bölükbaş and Öznurlu (2023) have reported that the telencephalon in chicks consists of seven regions, namely hyperpalium, mesopalium, nidopalium, arcopallium, striatum, pallidum and hippocampus. Gautam et al. (2020) state that the avian cerebral hemispheres consist of two parts: the dorsal pallium and ventral subpallium. The pallium is made up of the outer cortical area (hyperpallium/wulst) and the inner cortical area (dorsal ventricular ridge). Mesopallium, nidopallium and arcopallium are the lower parts of the dorsal ventricular ridge, and the striatum and pallidum parts form the subpallium (Abd‐Alrahman 2017; Abid and Al‐Bakri 2017). In birds, it is stated that the hyperpallium region of the dorsolateral corticoid area contains nerve fibres, whereas the hippocampus, amygdala and dorsal ventricular ridge contain pyramidal neurones (Abid and Al‐Bakri 2017). Although the pallium, which represents approximately 75% of the telencephalon in adult birds, establishes a connection between sensory and motor stimuli (Shepherd 1994), it is stated that the dorsal ventricular ridge receives somatosensory stimuli and transmits motor impulses to the basal ganglia (Skimizu 2009). Bain et al. (2007) mentioned a thin parenchyma structure, the thickening of the cortex and the shrinkage of the ventricle, which is relatively large in early developmental stages during incubation. Atallah et al. (2021) reported a homogeneous neuropil structure, and the cerebral cortex consisted of molecular, granular and pyramidal layers, the majority of which were formed by large triangular‐shaped pyramidal cells on the 20th day of incubation. Jarvis (2009) suggests that unlike the layered cortex structure of mammals, the bird brain has a structure that includes pallial (cortical‐like), striatal and pallidal regions. Although the striatal and pallidal regions of birds are more similar to those of mammals, it is also stated that these structures have similar functions and connections. Reiner, Yamamoto, and Karten (2005) reported that the bird telecephalon does not have a structure similar to the mammalian neocortex and resembles the basal ganglia in terms of the distribution of neurones. Data from this study were similar to previous studies.

When conducting stereological studies, it is essential to maintain an error coefficient value of 5% or less, particularly in volume measurements. If the value exceeds this limit, it is necessary to increase the number of sections and points counted (Selçuk and Tıpırdamaz 2020). In the current study, all coefficient of error (CE) values were found to be less than 0.05.

## 5 Conclusion

Mammalian and avian brains, which have physiologically similar functions and connections, are separated from each other by a histologically different cortex structure. Chick embryos, which are developmentally similar to human embryos, can be easily manipulated, and do not have a placental barrier, are frequently used as an important material in developmental embryonal studies as well as in neurotoxicity and embryotoxicity studies. Regional volume changes and neuronal losses are very important criteria for the prognosis of brain diseases resulting from neuronal degeneration and synaptic losses. Volumetric and cellular changes that occur in the neural tissue during the embryonic development of the brain are important data in the clinical identification and progression of brain diseases caused by endogenous or exogenous factors. We believe that the fact that both anatomical and embryological current data obtained from this study can be used as reference data in neurotoxicity and embryotoxicity studies adds originality to this study.

## Author Contributions

**Muhammet Lutfi Selcuk**: conceptualization, methodology, investigation, writing–original draft. **Fatma Kayikci**: conceptualization, methodology, writing–review and editing.

## Ethics Statement

Ethical approval was taken from the Ethical Committee of Health Sciences of Karamanoglu Mehmetbey University (protocol number: 2022/03).

## Conflicts of Interest

The authors declare no conflicts of interest.

### Peer Review

The peer review history for this article is available at https://publons.com/publon/10.1002/vms3.70124.

## Data Availability

The authors declare that data supporting the study findings are also available to the corresponding author.
